# Design and Implementation of Charge Pump Phase-Locked Loop Frequency Source Based on GaAs pHEMT Process

**DOI:** 10.3390/s22020504

**Published:** 2022-01-10

**Authors:** Ranran Zhao, Yuming Zhang, Hongliang Lv, Yue Wu

**Affiliations:** Key Laboratory of Wide Band-Gap Semiconductor Materials and Devices, School of Microelectronics, Xidian University, Xi’an 710071, China; zrr_8930@163.com (R.Z.); zhangym@xidian.edu.cn (Y.Z.); k3wwyk3wwy@126.com (Y.W.)

**Keywords:** charge pump phase-locked loop, GaAs pHEMT, low phase noise

## Abstract

This paper realized a charge pump phase locked loop (CPPLL) frequency source circuit based on 0.15 μm Win GaAs pHEMT process. In this paper, an improved fully differential edge-triggered frequency discriminator (PFD) and an improved differential structure charge pump (CP) are proposed respectively. In addition, a low noise voltage-controlled oscillator (VCO) and a static 64:1 frequency divider is realized. Finally, the phase locked loop (PLL) is realized by cascading each module. Measurement results show that the output signal frequency of the proposed CPPLL is 3.584 GHz–4.021 GHz, the phase noise at the frequency offset of 1 MHz is −117.82 dBc/Hz, and the maximum output power is 4.34 dBm. The chip area is 2701 μm × 3381 μm, and the power consumption is 181 mw.

## 1. Introduction

With the continuous development of integrated circuit technology, phase-locked loop (PLL) frequency source technology is widely used in various sensors, such as for high-accuracy clock generators for image sensors [1,2,3,4]. In the recent years, high accuracy sensors that have been extensively studied, especially for implantable medical sensors and high accuracy image sensors, require low power consumption, high output power, and low phase noise [5]. As the key module of sensors, the performance of the PLL deter-mines the performance of the sensor to a certain extent. Charge pump phase-locked loop (CPPLL) is a representative structure of PLL because of its low phase noise, variational phase difference, and high-frequency operation [6,7,8].

Numerous research results of CPPLL have been published, such as [9,10,11,12,13,14]. In [11], a CPPLL is realized using a 65 nm Si CMOS process. The proposed CPPLL employs a novel ultralow-voltage charge pump. The operating frequency of the proposed CPPLL is 0.09 GHz–0.35 GHz, the phase noise is −90 dBc/Hz at the frequency offset at 1 MHz, and the circuit consumes about 0.109 mW. In [9], a PLL based on GaAs pHEMT is proposed. The proposed PLL is optimized with a combination of several circuit techniques to reduce phase noise and increase the operation speed. The operating frequency of the proposed PLL is about 37 GHz, the phase noise is −98 dBc/Hz at a frequency offset of 1 MHz, and the circuit consumes about 480 mW. It can be seen from the above references that GaAs pHEMT has the characteristics of high gain, excellent power characteristics, and low noise [15,16,17]. The GaAs pHEMT process can be used to achieve low noise, higher output power PLL, but circuits based on the GaAs pHEMT process introduce a large power consumption while achieving higher frequencies, and there are many difficulties in designing CPPLLs based on the GaAs pHEMT process. In addition, the design of the CPPLL needs to compromise in terms of performance issues, such as phase noise, power consumption, area, and process. Therefore, this paper proposes an improved structure CPPLL based on a 0.15 µm GaAs pHEMT process. The proposed structure achieves a low phase noise and a high output power. The tradeoff between the area and power consumption has also been obtained.

## 2. Design of CPPLL

**Figure 1:**
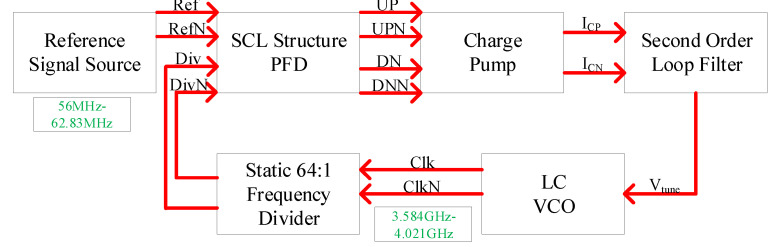
The system architecture of CPPLL.

The block diagram of the proposed CPPLL is presented in Figure 1. It consists of a SCL structure phase frequency detector (PFD), a charge pump (CP), a second order loop filter, a voltage-controlled oscillator (VCO), and a static 64:1 frequency divider (FD). Descriptions of the individual building blocks follow.

### 2.1. Phase Frequency Detector (PFD)

**Figure 2:**
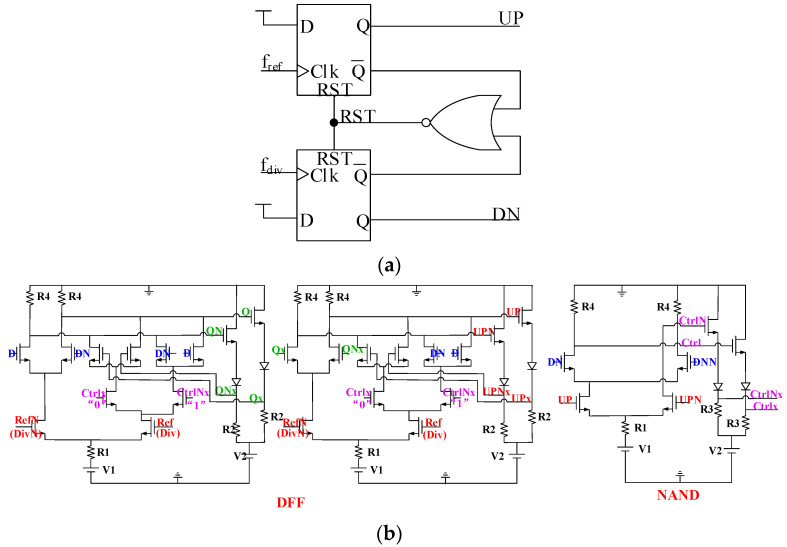
The structure of PFD: (**a**) the architecture of PFD; (**b**) the structure of PFD.

The phase frequency detector (PFD) can make the frequency of the reference signal and the frequency of the feedback signal of the FD equal, and can eliminate the phase difference between the two [18]. The structure of the PFD is shown in Figure 2, which consists of two D flip-flops (DFF) and one NAND reset circuit. The advantages of the PDF based on DFF structure are that it has a simple structure and low power consumption, and the theoretical phase detection range is very wide ([−2π, 2π]), which can an solve the problem of the phase detection dead zone in PFD.

The PFD adopts a low-threshold voltage depletion transistor, which can still detect the phase under low voltage conditions without using a delay unit to reduce the dead zone and increase the phase discrimination range. However, it also introduces the problem of logic errors when the latch is in the holding state. The level conversion circuit is used to pull down the level value of the feedback path, and under the condition of a low threshold voltage, the logic of the maintenance module is correct. Ref and RefN are reference signals, Div and DivN are differential feedback signals of FD, Ctrlx and CtrlNx are control signals outputted by NAND. If UP and DN are both “1”, NAND though Ctrlx and CtrlNx to make the reset module work, replacing the hold module in the latch, and making UP and DN output “0”. Otherwise, the reset model stops working, and the hold module is determined to work only by the input clock signal. The proposed PFD is based on a simple DFF structure which realized by all N-channel transistors, because the proposed structure does not contain delay units and the all N-channel transistors have lower power consumption, the PFD can realize lower power. Moreover, the maximum operating frequency (*f*_max_) of PFD is related to the reset delay time (*t*_reset_), the relationship is *f*_max_ ≤ 1/2 *t*_reset_, and the reset delay time of the latch depends on the parasitic capacitance. The parasitic capacitance can be decrease by reducing the size of the transistor, so that to achieve faster speed. In addition, the proposed PFD is a fully differential structure, which can better suppress common mode noise.

**Figure 3:**
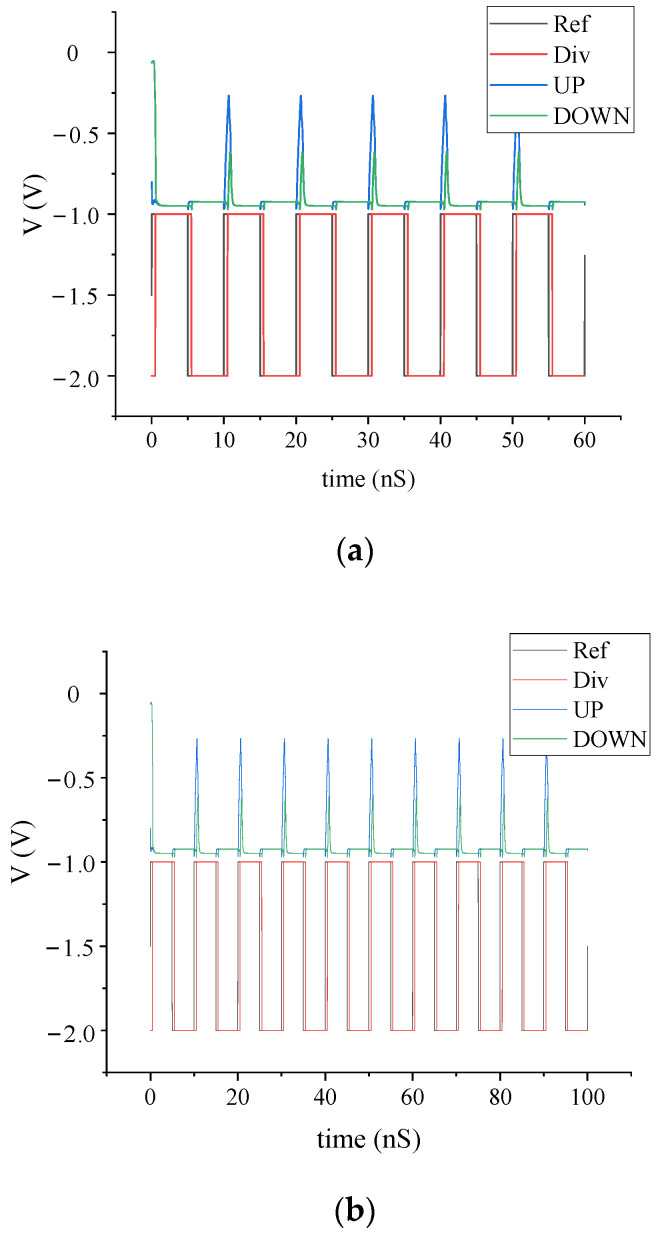
The simulation results of PFD: (**a**) the result of dead time; (**b**) the result of the maximum operating frequency.

In the proposed PFD, V1 = −1 V, V2 = −3 V, R1 = 10 Ohm, R2 = 120 Ohm, R3 = 94 Ohm, and R4 = 1391 Ohm. The simulation results of PFD are shown in Figure 3. The range of phase detector is [−355°, 355°], the power consumption of the core circuit is 5 mW, the dead time is (−5°, 5°), and the maximum operating frequency is 500 MHz. The reset delay time is 1 ns.

### 2.2. Charge Pump (CP)

**Figure 4:**
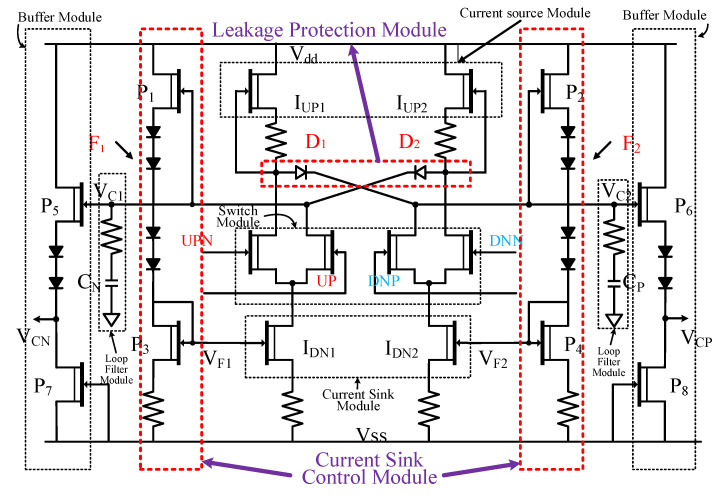
The structure of CP.

The charge pump (CP) is the core module of a PLL, and the bridge of digital signal and analog signal conversion [19]. A compound CP based on traditional structure is difficult to achieve in a steady state. In this paper, an innovative current sink control and leakage protection in steady-state technologies are proposed. The structure of the differential CP with a loop filter is shown in Figure 4.

The proposed CP based on steady-state protection and current sink control technology consisted of four parts, the current sink and current source module, switch module, current sink control module, and leakage protection module. The loop filter is composed of CN and CP. One port is grounded and the other is connected to the input of the buffer modules (P5 and P7, P6 and P8). The leakage protection module is composed of D1 and D2. When the input of CP is “1001”, because I_DN1_ ≥ I_UP1_ and I_DN2_ ≥ I_UP2_, and the unilateral conduction of D1 and D2, switch modules UPN and DNN are closed, and the current of current source module (I_UP1_ and I_UP2_) flow to the current sinks (I_DN1_ and I_DN2_). Therefore, leakage protection in the steady-state technique can prevent the leakage behavior of the capacitor in the loop filter for the current sink module. The charge pump can also appear in a steady state when the current source and current sink do not match. The current sink control module is composed of P1~P4. When V_C1_ increases, the current of I_UP2_ and the gate voltage (V_F1_) of I_DN1_ decreases, and the current of I_DN1_ increase. When V_C2_ increases, the current of I_UP1_ and the gate voltage (V_F2_) of I_DN2_ increases, and the current of I_DN2_ decrease. According to the above analysis, the current sink control module can ensure that the currents of I_DN1_ and I_DN2_ change with the currents of I_UP1_ and I_UP2_, respectively. The current sink control technique can reduce the requirement of accurate matching between the current sink and current source. As long as the current of the current sink is greater than or equal to that of the current source, which reduces the difficulty of the design and improves the implement ability of the CP, this permits the output control voltage to remain unchanged when the loop is locked, which meets the demand of the CPPLL.

**Figure 5:**
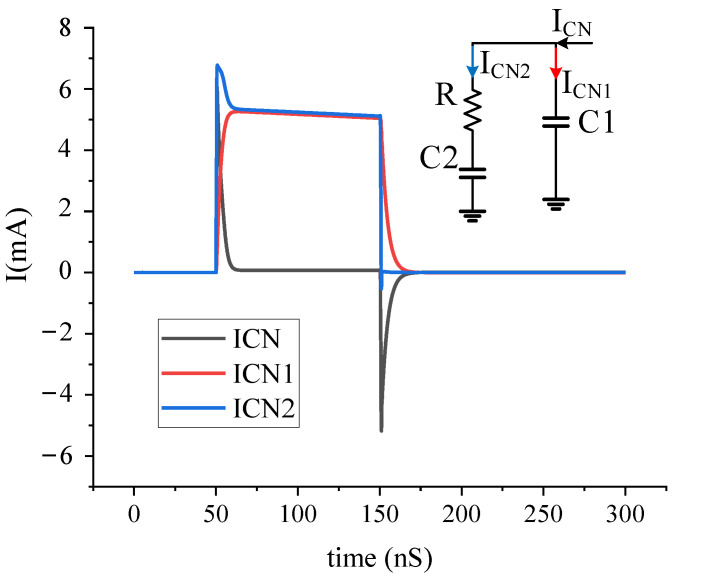
The simulation result of the ICN of the loop filter.

As shown in the Figure 5, the simulation results of the charge pump and loop filter show that the leakage protection module can effectively prevent charge leakage and maintain the control voltage unchanged. The I_CN_ of the charge pump is 5 mA, the static power consumption is 56 mW, and the area is 530 μm × 743 μm.

### 2.3. Voltage-Controlled Oscillator (VCO)

**Figure 6:**
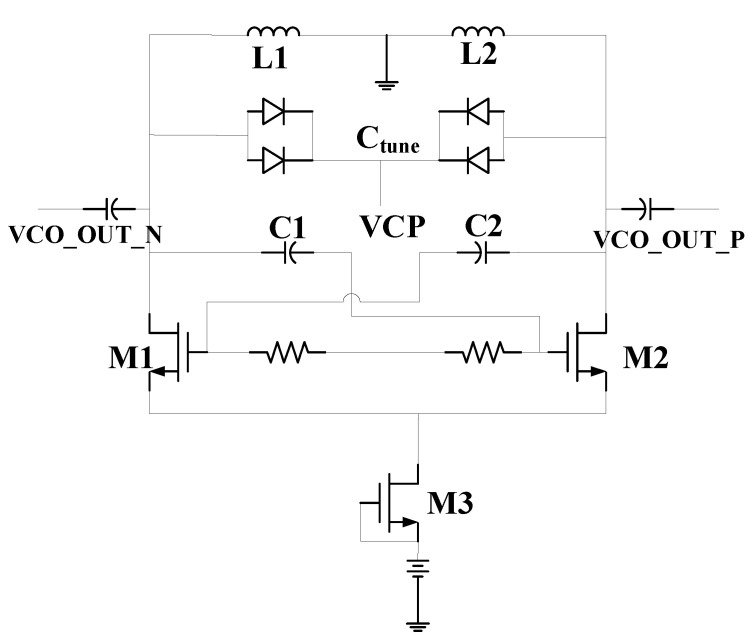
The structure of the VCO.

The voltage-controlled oscillator (VCO) is a circuit in the PLL that produces frequency changes through the voltage control. The phase noise and frequency tuning range are the two most important performance factors of the VCO [20,21]. In this paper, the cross-coupling structure VCO is adopted. The structure of the VCO is shown in Figure 6.

The VCO based on cross-coupling structure is a differential structure, the LC resonant circuit consists of the inductance (L1 and L2), a fixed capacitor (C1 and C2), the gate-drain capacitance of M1 and M2 (C_gd_), and a variable capacitor array (C_tune_). M1 and M2 provide G_m_ as negative resistance to compensate for the loss of the LC resonant circuit, which with a common source-amplifier configuration. The resonant circuit can be equivalent to two identical sub-resonant circuits and M1 and M2 provide energy for the two sub-resonant circuits to maintain oscillation through the cross-coupling structure. The differential structure can eliminate the influence of the common mode noise [22,23], and the swing amplitude of the output signal is:

(1) $$A_{diff}=\frac{4}{\pi }I_{bias}R_{t}$$

The amplitude of the VCO output signal is positively correlated with the tail current of the circuit. According to the Lessen phase noise formula, when other parameters are constant, the larger the amplitude of the output signal, the smaller the phase noise. GaAs pHEMT devices can work at higher voltages and currents. M3, as the tail current source device, can increase the amplitude of the VCO output signal. The size of the capacitor inductor will affect the quality factor of the circuit and the resonant frequency of the VCO. The size of the transistor will affect the output power and power consumption of the VCO. These parameters restrict each other so it is necessary to carefully design the size parameters of each device.

**Figure 7:**
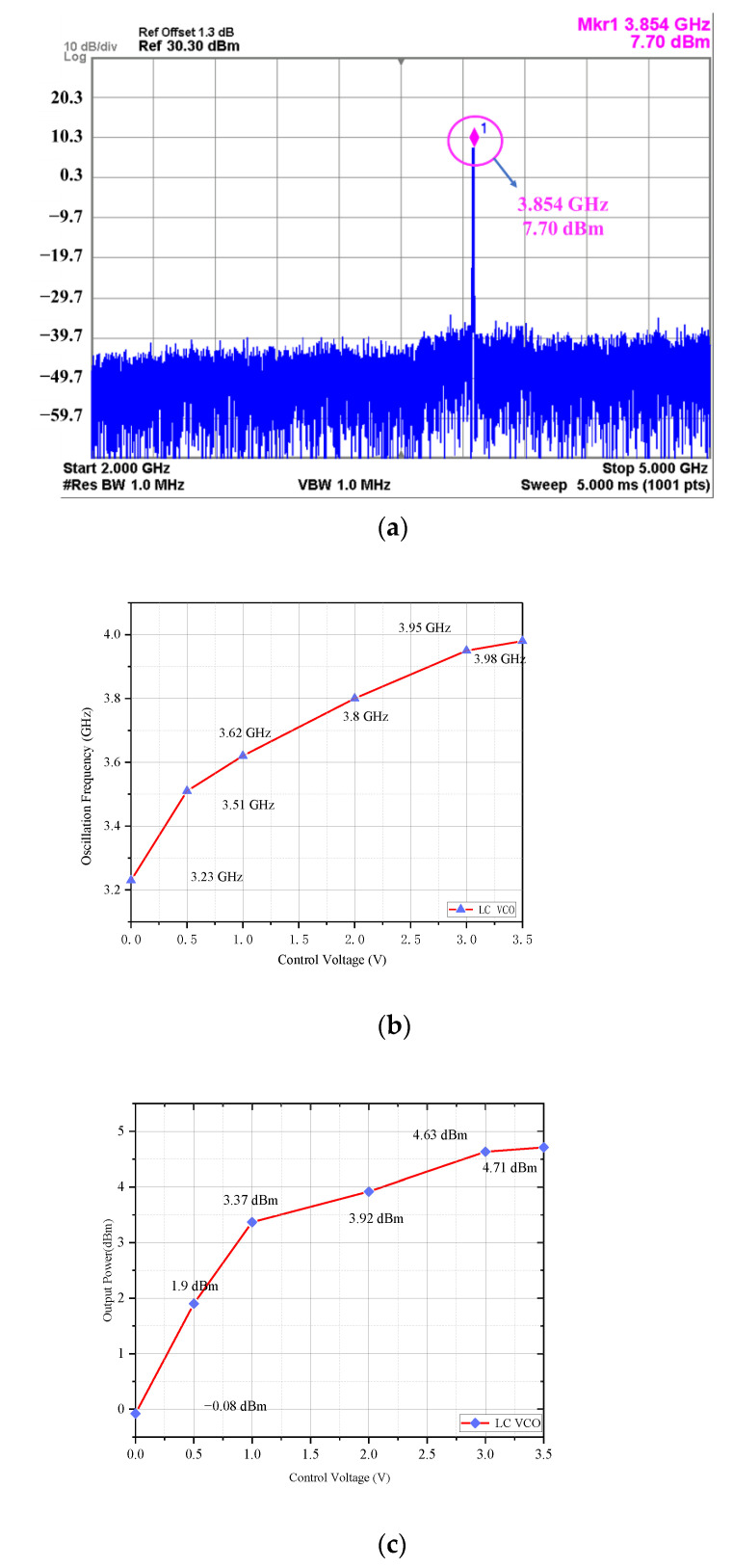
The measurement results of the VCO: (**a**) the spectrum of the VCO; (**b**) the tuning range of the VCO; (**c**) the output power of the VCO.

The measurement results of the VCO are shown in Figure 7. The results show that when the control voltage changes from 0 V to 3 V, the tuning range is 0.72 GHz, and the output oscillation frequency is 3.23 to 3.95 GHz. When the control voltage is 2 V, the phase noise of the VCO is −112.78 dBc/Hz at 1 MHz. The power consumption is 81 mW and the area is 1.016 mm × 0.614 mm.

### 2.4. Frequency Divider (FD)

**Figure 8:**
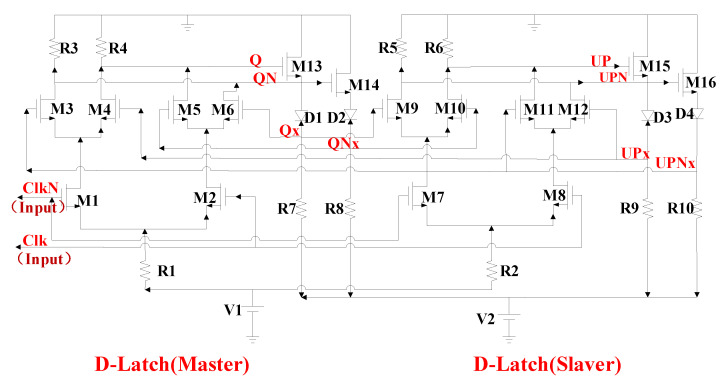
The structure of the static 2:1 FD.

In general, the signal frequency generated by the self-excited oscillation of the VCO will be relatively high, but the working frequency of the PFD is fairly low. This requires the FD to reduce the high-frequency signal and transfer it to the PFD. This paper presents a low-phase noise static 2:1 FD based on an improved SCL structure. The structure of the static 2:1 FD is shown in Figure 8. The maximum operating frequency of the flip-flop is:

(2) $$f_{MAX}=\frac{1}{2\tau }$$

*τ* is the delay time of the latch. Reducing the size of the transistor can increase the operating frequency of the FD, but it is possible that this will result in the working band not covering the low frequencies. In order to expand the working bandwidth of the FD, it is very important to reasonably design the size of each transistor.

**Figure 9:**
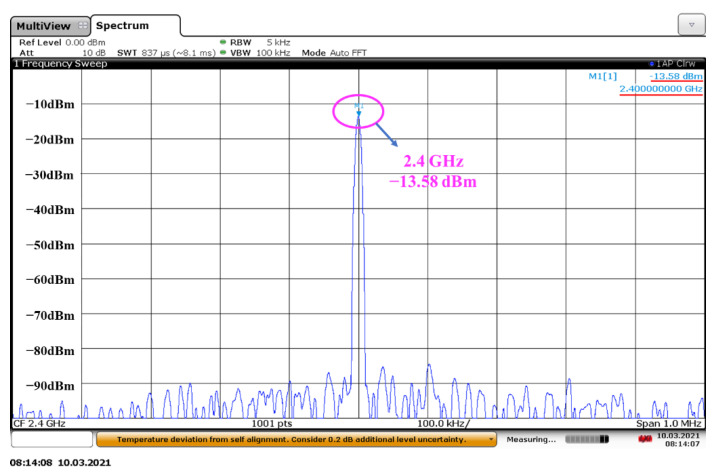
The test results of the FD: the frequency spectrum at input of 4.8 GHz.

The test results of the FD chip show that when the input signal frequency changes in the frequency range of 0–4.8 GHz. Since the lowest operating frequency of the FD can be close to that of DC, it is possible to achieve a 64:1 FD via direct cascading. The simulation results of FD are shown in Figure 9. The results show that the 64:1 FD can realize the frequency division function in the frequency range of 0–4.8 GHz.

## 3. Measurement Result and Discussion

**Figure 10:**
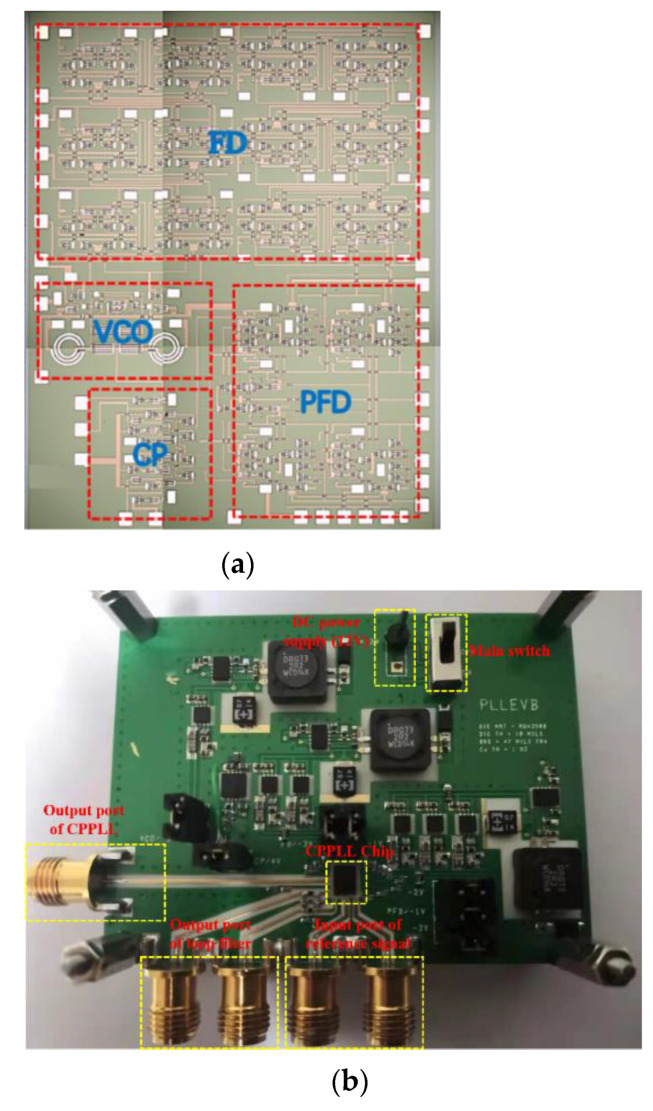
(**a**) Micrograph of the proposed CPPLL; (**b**) the test board.

The CPPLL was realized based on the Win PL 15-12 GaAs pHEMT process. Prudent symmetrical layout techniques are used to reduce the phase noise. A micrograph of the proposed CPPLL chip is shown in Figure 10a, and the chip area without PAD is 2.7 mm × 3.4 mm. In order to facilitate the test, the CPPLL test board was manufactured and a photo is shown in Figure 10b. The voltage required by the DC PAD is supplied by the low-voltage differential linear regulator LDO.

**Figure 11:**
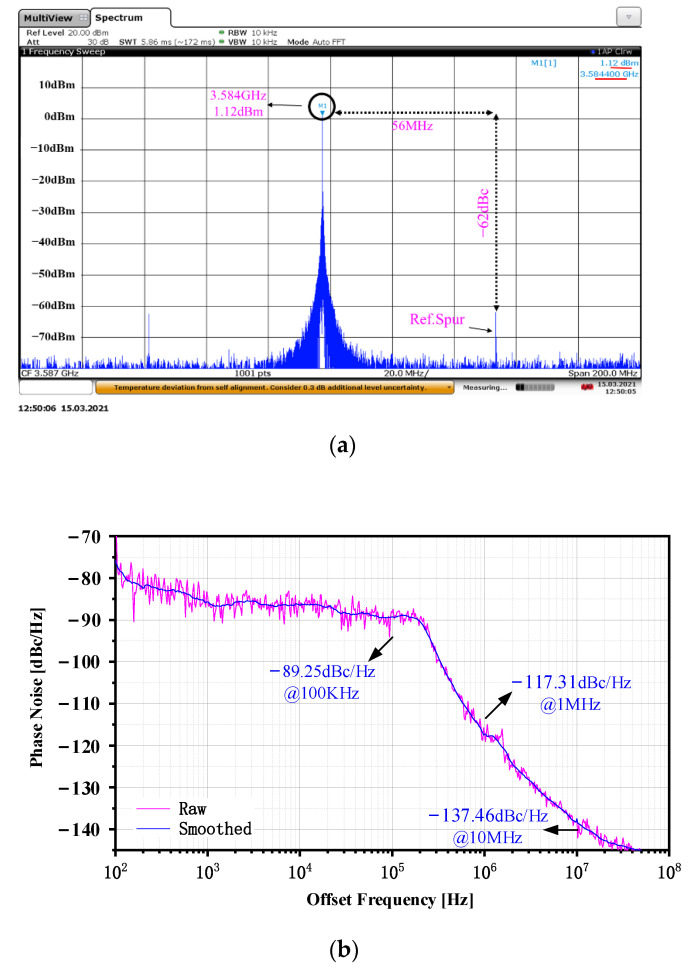
The test results when the output signal is 3.584 GHz: (**a**) the frequency spectrum; (**b**) the phase noise.

**Figure 12:**
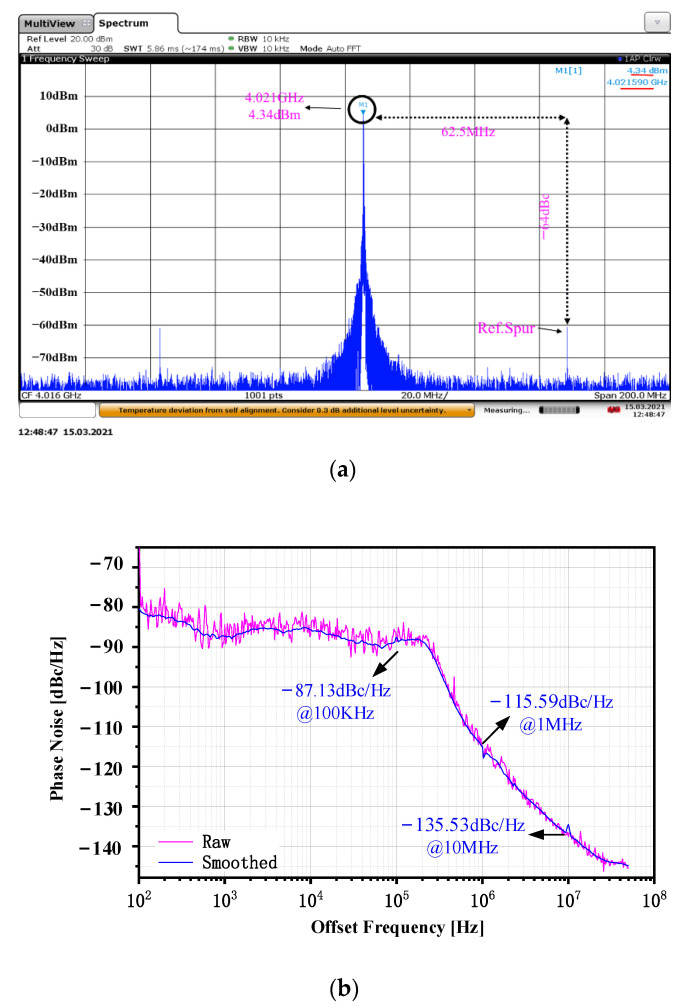
The test results when the output signal is 4.021 GHz: (**a**) the frequency spectrum; (**b**) the phase noise.

**Figure 13:**
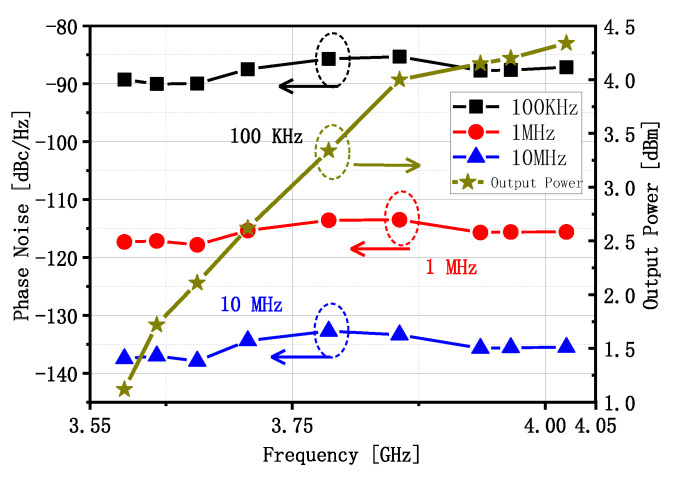
The phase noise and output power when the output signal frequency is 3.584 GHz to 4.021 GHz.

The test result shows that the operating frequency of the CPPLL is 3.584 to 4.021 GHz, and the bandwidth is about 0.44 GHz. Figure 11a shows the frequency spectrum when the output signal frequency is 3.584 GHz. The reference frequency is 56 MHz and the reference spur is −62 dBc. From the frequency spectrum and spurious signals it can be seen that the proposed CPPLL is locked. Figure 11b shows the phase noise when the output signal frequency is 3.584 GHz. The phase noise at a frequency offset of 100 kHz, 1 MHz, and 10 MHz are −89.25, −117.31, and −137.46 dBc/Hz. Figure 12a shows the frequency spectrum when the output signal frequency is 4.021 GHz. The output signal power is 4.34 dBm, the reference frequency is 62.5 MHz, and the reference spur is −64 dBc; the CPPLL is also locked. Figure 12b shows the phase noise when the output signal frequency is 4.021 GHz. The phase noise at a frequency offset of 100 kHz, 1 MHz, and 10 MHz are −87.13, −115.59, and −135.53 dBc/Hz. Figure 13 shows the phase noise and output power when the output signal frequency is 3.584 GHz to 4.021 GHz. The minimum phase noise when the frequency offset is 1 MHz is −117.82 dBc/Hz and the maximum output power is 4.34 dBm.

The performance of the proposed CPPLL is summarized and compared with some previous works, as shown in Table 1. The proposed CPPLL demonstrates a wide bandwidth, higher output power, lower phase noise, and is more suitable for high-accuracy implantable medical sensors and image sensor use.

## 4. Conclusions

In this paper, a charge pump phase locked loop (CPPLL) frequency source circuit is proposed. Through theoretical analyses, the methods of reducing the dead zone of the PFD and an innovative current sink control and leakage protection in the steady-state technologies of CP are proposed. In addition, a low-noise voltage-controlled oscillator (VCO) and a static 64:1 frequency divider are realized. The proposed CPPLL is realized by cascading each module based on a 0.15 µm GaAs pHEMT process. Measurement results show that the operating frequency of the proposed CPPLL is 3.584 GHz–4.021 GHz, and the consumed power is 181 mW. The lowest phase noise is −131.06 dBc/Hz@1MHz, and the maximum output power is 4.34 dBm. The chip area is 2701 μm × 3381 μm. In this paper, compound semiconductor technology and CPPLL technology are combined to verify the feasibility of a compound semiconductor process designed CPPLL, which effectively improves the circuit noise and increases the operating frequency and output power. The proposed CPPLL can be used as a local oscillator frequency source in 5G communication transceivers to provide local oscillator signals.

## Figures and Tables

**Table 1:** Comparison with some previously reported CPPLLs.

Ref.	Tech.	Structure	Freq. Range (GHz)	Phase Noise (dBc/Hz)	P_OUT_ (dBm)	P_DC_ (mW)	Area (mm^2^)
[11]	65 nm Si CMOS	CPPLL	0.09–0.35	−90@1MHz	−8	0.109	0.0081
[12]	65 nm Si CMOS	CPPLL	2.4	−122@1MHz	−2.25	1.02	0.3
[9]	0.2 μm GaAs pHEMT	APLL	37	−98@1MHz	-	480	1.7
This Work	0.15 μm GaAs pHEMT	CPPLL	3.584–4.021	−117.8@1MHz	4.34	39.69	2.7 × 3.4

## Data Availability

All data included in this study are available upon request by contact with the corresponding author. The data are not publicly available due to no available online server being available for the research group temporarily.
